# Binding of Natural Inhibitors to Respiratory Complex I

**DOI:** 10.3390/ph15091088

**Published:** 2022-08-31

**Authors:** Jonathan Schiller, Volker Zickermann

**Affiliations:** 1 Institute of Biochemistry II, University Hospital, Goethe University, 60438 Frankfurt am Main, Germany; 2 Centre for Biomolecular Magnetic Resonance, Institute for Biophysical Chemistry, Goethe University, 60438 Frankfurt am Main, Germany

**Keywords:** mitochondria, respiratory chain, NADH dehydrogenase, Parkinson’s disease, rotenone, piericidin, acetogenin

## Abstract

NADH:ubiquinone oxidoreductase (respiratory complex I) is a redox-driven proton pump with a central role in mitochondrial oxidative phosphorylation. The ubiquinone reduction site of complex I is located in the matrix arm of this large protein complex and connected to the membrane via a tunnel. A variety of chemically diverse compounds are known to inhibit ubiquinone reduction by complex I. Rotenone, piericidin A, and annonaceous acetogenins are representatives of complex I inhibitors from biological sources. The structure of complex I is determined at high resolution, and inhibitor binding sites are described in detail. In this review, we summarize the state of knowledge of how natural inhibitors bind in the Q reduction site and the Q access pathway and how their inhibitory mechanisms compare with that of a synthetic anti-cancer agent.

## 1. Introduction

Complex I of the respiratory chain is a 1 MDa membrane protein complex with 44 different subunits in mammals [1,2,3]. The enzyme transfers electrons from NADH to ubiquinone (Q) and uses the redox energy to drive vectorial proton translocation across the inner mitochondrial membrane. With a pump stoichiometry of 4H^+^/2e^−^, it contributes about 40% of the proton motive force (pmf) that powers ATP synthase. Under specific conditions, i.e., in the presence of a strong pmf and a reduced Q pool, complex I can run in reverse and transfer electrons from reduced ubiquinone (QH2) to NAD^+^.

Complex I has a central function in metabolism because of the following: (i) it regenerates oxidized NAD^+^, which is required for essential dehydrogenase reactions, e.g., in the citric acid cycle; and (ii) it is the entry point for electrons into the respiratory chain, which is central to oxidative phosphorylation. Therefore, mutations in the genes of complex I subunits or assembly factors cause a broad range of neuromuscular and neurodegenerative diseases [4,5,6,7]. In addition, oxygen radical formation during reverse electron transfer causes reperfusion injury in stroke and myocardial infarction [8].

**Figure 1:**
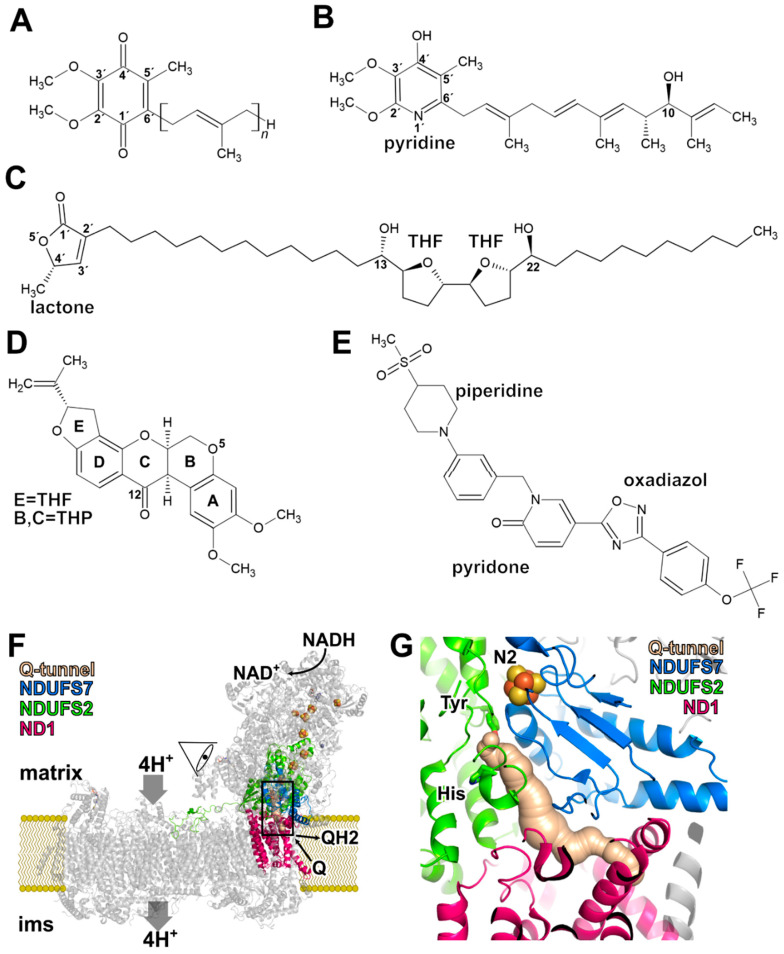
Complex I inhibitors and structural basis of Q binding and reduction. Chemical structures of (**A**) ubiquinone (Q10, *n* = 10), (**B**) piericidin A, (**C**) an annonaceous acetogenin, (**D**) rotenone, and (**E**) IACS-2858 (THF = tetrahydrofuran ring, THP = tetrahydropyran ring). (**F**) Architecture of respiratory complex I with NDUFS7 (blue), NDUFS2(green), and ND1 (pink), and the Q tunnel. The viewpoint is indicated and the section of the detail view in Figure 2 and Figure 3 is highlighted by a box. (**G**) Detail view of the Q reduction site and access pathway as shown in Figure 2 and Figure 3; a tyrosine and a histidine residue of NDUFS2 play a key role for binding the Q head group (compare Figure 2, residue numbers see Table 1, for details see text).

The structure of complex I has been determined by X-ray crystallography and cryo-EM (reviewed in [2,3,9]). However, despite the surge in structural information, the catalytic mechanism of complex I is debated. Complex I consists of a hydrophilic arm projecting into the mitochondrial matrix and a hydrophobic arm embedded in the inner membrane of the mitochondrion. Redox chemistry occurs exclusively in the matrix arm, whereas proton pumping activity is assigned to the membrane arm (Figure 1). In the matrix arm, electrons are transferred from the initial electron acceptor FMN through a chain of FeS centers to the site of ubiquinone reduction at the FeS center N2, which is located 20 Å above the membrane surface. This is an unusual topology considering that the electron acceptor ubiquinone is a very hydrophobic molecule with a long side chain of typically 10 isoprenoid units (Q10) (Figure 1A). A 35 Å long tunnel permits the transfer of the hydrophobic molecule from the membrane into the matrix arm (Figure 1F,G) [10]. When the Q head group binds in the vicinity of cluster N2, nearly the complete isoprenoid chain is inserted into the tunnel [11,12]. Interestingly, the tunnel is not entirely hydrophobic but is interrupted by a charged segment. This unusual topology should ensure that Q10 is not too strongly retained by hydrophobic interactions in the tunnel, and it was demonstrated that Q10 dissociation is not rate limiting for complex I [11]. The dynamics of Q in the tunnel have been investigated by molecular simulations and up to five different Q-binding sites were identified [13,14,15]. Residues important for Q binding were initially identified by site-directed mutagenesis (reviewed in [16]). Several X-ray and cryo-EM structures include Q molecules [12,17,18,19,20,21,22], and the binding mode of Q in the active site and in the access pathway is now known in considerable detail (reviewed in [23]).

For a long time, there has been a great interest in inhibitors of complex I [24,25]. Inhibitors from biological sources such as rotenone, piericidin A, and annonaceous acetogenins have IC50 values tested with bovine heart mitochondria in the low nanomolar range [26]. Their use in agriculture, fishing, fish farming, and traditional medicine has a long history in some cases (reviewed in [25,27,28,29]). However, the use of complex I inhibitors was restricted after evidence of an association with neurodegenerative diseases increased. In the 1980s, the pro-toxin MPTP (N-methyl-4-phenyl-1,2,3,6-tetrahydropyridine) was identified as the cause for acute symptoms indistinguishable from Parkinsons disease (reviewed in [30]). The active compound MPP^+^ (1-methyl-4-phenylpyridinium) is a complex I inhibitor that accumulates in dopaminergic neurons by uptake of the dopamine transporter. After the link of nigrostriatal degeneration with complex I inhibition was recognized, attempts were made to develop rotenone-based animal models for Parkinson’s disease (reviewed in [31]). More recent medical applications of compounds acting on complex I include their use against diabetes [32,33], selected types of cancers [34,35,36,37], and to counteract reperfusion injury [38,39,40]. Mitochondria as a therapeutic target for common disease have been reviewed by Murphy and Hartley [41]. Complex I inhibitors continue to be indispensable tools to decipher the still unknown mechanism of coupling between redox chemistry and proton translocation [24]. Structures of inhibitor bound complex I have been determined by X-ray crystallography [10,17,42] and cryo-EM [18,21,43,44,45]. Here, we will focus on inhibitors from biological sources and review the interaction of rotenone, piericidine A, and an annonaceous acetogenin with mitochondrial complex I in comparison with a synthetic anticancer agent.

## 2. Rotenone

Rotenone is found in the roots and stems of *Lonchocarpus* and *Derris* species, such as *Derris eliptica*. Traditionally, rotenone-containing plant extracts were used by indigenous people for fishing. Its chemical structure (Figure 1) was reported in 1933 [46] and was identified as a complex I inhibitor almost 30 years later [47]. Rotenone comprises a five-ring structure (Figure 1). The three rings A, C, and D form the basic isoflavone structure. Ring A carries two methoxy groups and thus shows similarity with the ubiquinone head group. Rotenone belongs to a family of similarly structured isoflavonoids with inhibitory activity [48] that can be isolated from different plant species. Rotenone is severely toxic to insects and aquatic vertebrates but exhibits comparatively less acute toxicity to mammals when ingested orally (LD50 for mice 350 mg/kg for oral uptake and 2.8 mg/kg for intravenous application, reviewed in [28]). While rotenone is efficiently metabolized in the human gut, fish absorb rotenone through the gills directly into the blood stream [28]. Because of these properties it was widely employed in agriculture and fish farming. Its use has been discontinued due to safety concerns after evidence of a link between complex I inhibitors and neurogenerative diseases accumulated. Betarbet et al. showed that systemic exposure of rats to subacute toxic doses of rotenone caused highly selective degeneration of dopaminergic neurons in the substantia nigra with accumulation of characteristic cytoplasmic inclusions containing α-synuclein [49]. However, there is mounting evidence that complex I inhibition is only one factor in the pathogenesis of rotenone-induced Parkinsonism. It was shown as early as 1974 that rotenone interferes with the dynamics of microtubule formation [50], and dysregulation of the cytoskeleton is thought to be a considerable factor for the death of neurons [51,52]. The pronounced vulnerability of dopaminergic neurons might be caused by high intracellular ROS levels due to increased activity of monoamine oxidase as a consequence of impaired microtubule-associated transport of dopamine-containing vesicles [51]. Increased ROS levels might also be caused or exacerbated by rotenone-stimulated ROS release from NADPH oxidase of microglial cells [53].

## 3. Piericidin A

The piericidins form a family of compounds that are produced by actinomycetes of the *Streptomyces* or *Nocardioides* genera [54]. A striking example for the antibiotic action of these compounds is the symbiosis of beewolf digger wasps with streptomyces species that are incorporated into the larval cocoon [55]. The bacteria produce a cocktail of substances including several piericidins that lead to a protection of the wasp offspring against pathogens. Piericidin A was discovered as an insecticide in 1963 [56] and was later identified as an inhibitor of respiratory complex I [57]. Piericidin A (Figure 1) is a pyridine derivative substituted with 2′, 3′ methoxy groups, a 4′ hydroxy group, and a 5′ methyl group. In position 6′, the pyridine ring carries a side chain with a hydroxy group. Obviously, the overall structure of piericidin A bears close similarity to ubiquinone (Figure 1A,B) but with differences in both the head group and the side chain. Scatchard analyses provided evidence for two binding sites in complex I [58] and structure activity relationship (SAR) studies showed that the 4′ hydroxy, the 5′ methyl group and the structure of the side chain are important for inhibitory potency (reviewed in [54]). Interestingly, piericidin A exposure of transgenic mice carrying the P301S mutation in the tau gene caused aggravation of hereditary tau pathology [59]. In contrast to this link of piericidin A with neurodegeneration, it was shown that this inhibitor does not induce selective loss of dopaminergic neurons in test animals questioning a specific role of complex I inhibition in Parkinson’s disease [52].

## 4. Annonaceous Acetogenins

Acetogenins are members of a large family of compounds derived from plants of the *Annonaceae* family like *Annona muricata* [27]. Native to tropic regions of America, *A. muricata* can now be found in tropical and subtropical regions around the world. Acetogenins are characterized by a long aliphatic chain with an α, β-unsaturated γ-lactone ring and up to three tetrahydrofuran (THF) rings (Figure 1C). They were shown to be complex I inhibitors [60], and the bis-tetrahydrofuran compounds rolliniastatin-1 and rolliniastatin-2 (=bullatacin) have stronger inhibitory potency than piericidin or rotenone [26]. Several annonaceous acetogenins are used in traditional medicine [27] and have been investigated for their potential use as anticancer agents [61,62]. However, toxic effects of annonaceous acetogenins have been implicated in the etiology of a sporadic tauopathy in Guadaloupe raising safety concerns for this group of compounds [63,64].

## 5. Binding of Q in the Active Site and in the Access Pathway Connecting It to the Membrane

The Q reduction site and the tunnel connecting it with the membrane are essentially formed by three subunits (Figure 1F,G). The hydrophobic ND1 subunit is part of the membrane arm and constitutes the initial part of the access pathway with a narrow portal consisting of two transmembrane helices and a short surface helix. In the matrix arm, the continuation of the tunnel and the active site itself is formed by the NDUFS2 and NDUFS7 subunits. At the interface of the three subunits, a section of the Q tunnel comprises several charged residues.

The immediate electron donor for Q reduction is the iron sulfur cluster N2, which is coordinated by four cysteine residues of the NDUFS7 subunit. A number of residues in the NDUFS7 and NDUFS2 subunits have been identified by mutagenesis as functionally significant for binding of ubiquinone and inhibitors [16,65]. From early on, a tyrosine and a histidine residue in NDUFS2 were the focus of interest as potential ligands of the Q head group [10,16]. These two residues are discussed as prime candidates to donate the protons required in Q redox chemistry. Together with further conserved residues, the histidine residue forms a loop connecting the first two strands of the N-terminal β-sheet of the NDUFS2 subunit. Conformational changes of this β1 β2 loop are thought to be mechanistically important [18,20,22,42]. The Q-binding pocket contains a conserved aspartate residue that was modified in ligand-directed tosyl chemistry (LDT) studies with an acetogenin-type inhibitor [66]. In many structures, the histidine residue in the β1 β2 loop and the aspartate residue are in hydrogen-bonding distance. Dynamic interaction of the two residues could play an important role in the coupling mechanism [67]. The tyrosine-histidine-aspartate triad and several other residues in the Q reduction site and in the Q tunnel are strictly conserved. Table 1 compiles a selection of residue numbers for complex I from different species.

**Figure 2:**
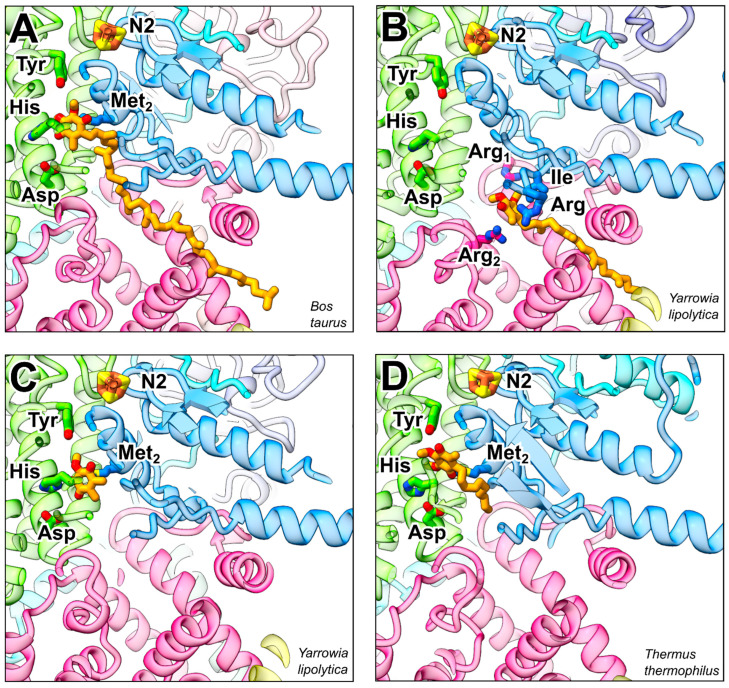
Q-binding sites in respiratory complex I. Q reduction site near FeS cluster N2 and access pathway for Q from the membrane with NDUFS7 (blue), NDUFS2 (green), and ND1 (pink) (compare Figure 1); Q molecules and residues discussed in the text are shown in stick representation (numbering see Table 1). (**A**) Q10 bound to complex I from *B. taurus* in lipid nanodisc (PDB ID 7QSK), (**B**) native Q9 in the Q access pathway of complex I from *Y. lipolytica* (PDB ID 6RFR), (**C**) head group of decyl benzoquinone (DBQ) bound to complex I from *Y. lipolytica* captured under turnover (PDB ID 7O6Y), and (**D**) DBQ bound to complex I from *T. thermophilus* (PDB ID 6I0D).

Several structures of Q bound complex I have been reported. There are structures containing Q molecules with long isoprene chains, either derived from the native membrane [19,21,68,69] or reconstituted [12], or those containing short-chain Q analogs [17,18,20,21]. Furthermore, structures can be distinguished in those where the head group is located in electron transfer distance to the cluster N2 and those in which the head group is located in intermediate binding sites in the access pathway. In the following, we focus on a few examples (Figure 2). The X-ray structure of *T. thermophilus* was solved with a short-chain Q analogue bound in the Q reduction site [10,17]. One of the carbonyl groups of the Q head group is bound to the tyrosine residue ($\mathrm{Tyr}87_{\mathrm{Tt}}^{\mathrm{FS}2}$). However, there is no close contact with the β1 β2 loop histidine ($\mathrm{His}38_{\mathrm{Tt}}^{\mathrm{FS}2}$), which is in hydrogen-bonding distance with the aspartate residue ($\mathrm{Asp}139_{\mathrm{Tt}}^{\mathrm{FS}2}$) (Figure 2D). Another Q-binding pose was observed for Q10 in a cryo-EM structure of mammalian complex I [12]. Here, the tyrosine residue ($\mathrm{Tyr}108_{\mathrm{Bt}}^{\mathrm{FS}2}$) is too distant for a direct hydrogen bond to the Q head group (Figure 2A), but a contact of a carbonyl group and the tyrosine hydroxyl group is mediated via two water molecules. In contrast to the corresponding structure of *T. thermophilus* complex I, the β1 β2 loop histidine ($\mathrm{His}59_{\mathrm{Bt}}^{\mathrm{FS}2}$) contacts the other Q carbonyl group and the adjacent methoxy group. The His-Asp distance is longer as compared with *T. thermophilus* complex I. Another contact is made with a conserved methionine residue ($\mathrm{Met}60_{\mathrm{Bt}}^{\mathrm{FS}7}$) of the NDUFS7 subunit. The side chain of this residue points to the center of the Q headgroup. Interestingly, two other methionine residues ($\mathrm{Met}152_{\mathrm{Bt}}^{\mathrm{FS}2}$, $\mathrm{Met}59_{\mathrm{Bt}}^{\mathrm{FS}7}$) are present in close distance and contact the head group and the side chain of Q. A cryo-EM structure of complex I from the yeast *Yarrowia lipolytica* captured under turnover conditions with decyl benzoquinone (DBQ) showed a comparable distance of the Q head group and the tyrosine ($\mathrm{Tyr}144_{\mathrm{Yl}}^{\mathrm{FS}2}$) as observed in mammalian complex I [20]. Likewise, a close contact of the β1 β2 loop histidine ($\mathrm{His}95_{\mathrm{Yl}}^{\mathrm{FS}2}$) with one of the methoxy groups of the Q head group was observed but there was no binding to the carbonyl group. Consistent with the situation in mammals, the methione residue ($\mathrm{Met}91_{\mathrm{Yl}}^{\mathrm{FS}7}$) points to the center of the ubiquinone head group (Figure 2C).

In another cryo-EM structure of complex I from the yeast *Yarrowia lipolytica* (Figure 2B), a native Q9 molecule was observed in the Q tunnel at the interface of NDUFS7 with ND1 [19]. The head group is situated in the charged region of the Q tunnel, with $\mathrm{Arg}36_{\mathrm{Yl}}^{\mathrm{ND}1}$, $\mathrm{Arg}297_{\mathrm{Yl}}^{\mathrm{ND}1}$, and $\mathrm{Arg}108_{\mathrm{Yl}}^{\mathrm{FS}7}$ coming closest but not forming hydrogen bonds or ion pairs. Conserved $\mathrm{Ile}106_{\mathrm{Yl}}^{\mathrm{FS}7}$ points to the center of the head group and was shown to be functionally important by site-directed mutagenesis [70]. This Q-binding site is interesting because it is situated at the entrance of the so-called E channel [10] leading into the membrane arm, and is therefore in a strategic position for coupling electron transfer to proton pumping [20].

Taken together, different binding modes of Q in or near the active site have been described, and critical residues for binding the Q head group have been identified. However, the hypothesized Tyr-Q-His arrangement for simultaneous proton transfer to the 1,4 carbonyl groups of the Q head group has not been observed yet in X-ray or cryo-EM structures. Conformational changes of the site may allow for the formation of such an arrangement as suggested by MD simulations [71]. Bound Q molecules were also observed in the access pathway to the active site. The significance of these binding sites for the mechanism is still unclear.

## 6. Binding Sites of Natural Inhibitors

**Figure 3:**
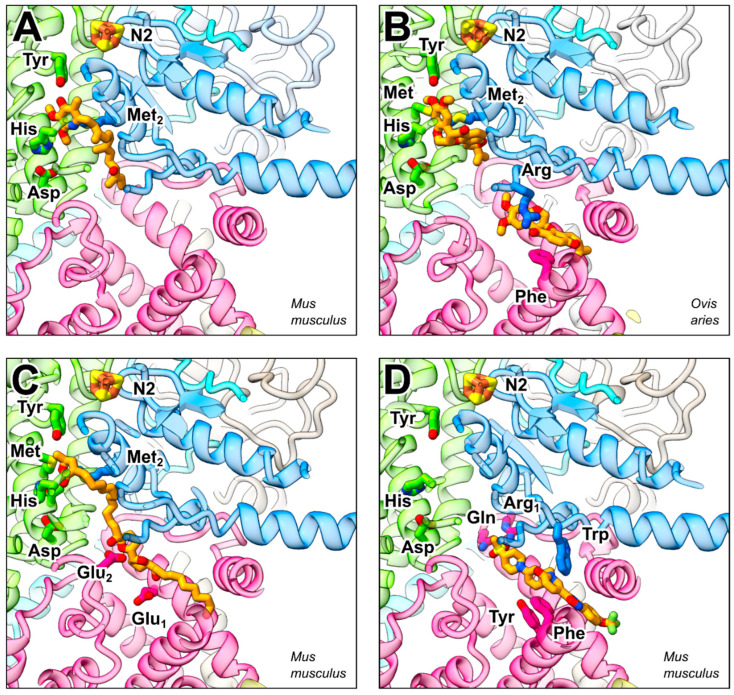
Binding of natural inhibitors and IACS-2858 to respiratory complex I. Q reduction site near FeS cluster N2 and access pathway as shown in Figure 2. (**A**) Piericidin A bound to complex I from *M. musculus* (PDB ID 6ZTQ), (**B**) two rotenone molecules bound to complex I from *O. aries* (PDB ID 6ZKN), (**C**) annonaceous acetogenin (compound 1 as described in Grba et al., 2022) bound to complex I from *M. musculus* (PDB ID 7PSA), and (**D**) the synthetic anti-cancer agent IACS-2858 bound to complex I from *M. musculus* (PDB ID 7B93); for residue numbering see Table 1.

The binding of piericidin in bacterial and mammalian complex I (Figure 3A) was studied by X-ray crystallography [17] and by cryo-EM [43], respectively. In both cases the conserved tyrosine residue ($\mathrm{Tyr}87_{\mathrm{Tt}}^{\mathrm{FS}2}$, $\mathrm{Tyr}108_{\mathrm{Mm}}^{\mathrm{FS}2}$) of the Q reduction site (Table 1) is in hydrogen-bonding distance with the 4′ hydroxyl group of the ring structure. In mammalian complex I, the β1 β2 loop histidine ($\mathrm{His}59_{\mathrm{Mm}}^{\mathrm{FS}2}$) forms a hydrogen bond with the piericidin 2′ methoxy group. This interaction appears to be weaker in the bacterial enzyme. Further residues involved in binding of piericidin are two methionine residues, one from FS2 ($\mathrm{Met}152_{\mathrm{Mm}}^{\mathrm{FS}2}$) and one from FS7 $(\mathrm{Met}70_{\mathrm{Mm}}^{\mathrm{FS}7}$). Only the latter residue is conserved and appears to be less important for binding of pieridin in *T. thermophilus*. There is no close contact of the pyridine ring nitrogen with the protein structure. Interestingly, the maps of the inhibitor-bound mammalian enzyme and MD simulations give some evidence for a second piericidin binding site in mammalian complex I [43]. In the charged region of the tunnel, the C10-hydroxyl of the side chain of a piericidin molecule bound as described above and the head group of a second molecule may form a hydrogen-bonding interaction. A second piericidin binding site in complex I is in agreement with initial inhibitor binding experiments [58].

The structural basis of rotenone binding has been elucidated in complex I from pig [21] and sheep [18]. Rotenone is a bulky molecule with a kink between the A/B and C/D/E ring systems (Figure 3B and Figure S1). It is still unclear how rotenone can pass the narrow opening of the Q tunnel. Conformational changes seem necessary even for the Q head group to pass through [72]. The A ring of rotenone occupies a position that overlaps with that of the Q head group bound in the Q reduction site (Figure 3B and Figure S1B). The tyrosine residue ($\mathrm{Tyr}108_{\mathrm{Oa}}^{\mathrm{FS}2}$) that binds the Q head group is in hydrogen-bonding distance to the oxygens of both methoxy groups of the A ring. One of the methyl groups is contacted by a methionine residue ($\mathrm{Met}152_{\mathrm{Oa}}^{\mathrm{FS}2}$) of NDUFS2. The β1 β2 loop histidine ($\mathrm{His}59_{\mathrm{Oa}}^{\mathrm{FS}2}$) sits between the five oxygen of the B ring and the aspartate residue ($\mathrm{Asp}160_{\mathrm{Oa}}^{\mathrm{FS}2}$). A contact of $\mathrm{Thr}156_{\mathrm{Oa}}^{\mathrm{FS}2}$ with the B ring seems to be important because a Ser to Thr exchange at this position substantially increased the moderate rotenone sensitivity of wild-type *Y. lipolytica* complex I [73]. The C/D/E rings are located in a pocket formed by three phenylalanine residues of NDUFS2 $(\mathrm{Phe}167_{\mathrm{Oa}}^{\mathrm{FS}2}$, $\mathrm{Phe}168_{\mathrm{Oa}}^{\mathrm{FS}2})$ and NDUFS7 ($\mathrm{Phe}76_{\mathrm{Oa}}^{\mathrm{FS}7}$), respectively, and two methionine residues of NDUFS7 ($\mathrm{Met}59_{\mathrm{Oa}}^{\mathrm{FS}7}$, $\mathrm{Met}60_{\mathrm{Oa}}^{\mathrm{FS}7}$). Interestingly, the C/D/E ring position superimposes nicely with the first two isoprenoid units of Q10 in the Q-bound high-resolution structure of bovine complex I [12] (Figure S1D). A second rotenone molecule (Figure 3B and Figure S1C) was discovered matching the position of a Q molecule in the Q access pathway [18]. An arginine residue ($\mathrm{Arg}77_{\mathrm{Oa}}^{\mathrm{FS}7}$) of NDUFS7 binds to the carbonyl oxygen in position 12, and a stacking interaction is present between a phenylalanine ($\mathrm{Phe}224_{\mathrm{Oa}}^{\mathrm{ND}1}$) side chain and the D ring. Surprisingly, a third rotenone molecule was found in a remote site in the membrane arm bound in the ND4 subunit coordinated by the residues $\mathrm{Arg}142_{\mathrm{Oa}}^{\mathrm{ND}4}$, $\mathrm{Lys}206_{\mathrm{Oa}}^{\mathrm{ND}4}$, and $\mathrm{Trp}215_{\mathrm{Oa}}^{\mathrm{ND}4}$ [18]. Interesting here is the lysine residue ($\mathrm{Lys}206_{\mathrm{Oa}}^{\mathrm{ND}4}$), which is part of a water wire traversing the whole membrane arm. This could be the reason for the ability of rotenone to inhibit the Na^+^/H^+^ antiport activity observed in deactivated mammalian complex I [74] (Figure S1E).

The binding of annonaceous acetogenins to complex I was elucidated using complex I from *M. muculus* (Figure 3C) [44]. The compound used in this study (Figure 1C) is very similar to the naturally occurring bullatacin. Regarding its interaction with complex I, there are striking differences to the previously described inhibitors piericidin and rotenone. The acetogenin has a length with which it fills the entire Q tunnel from the entrance to the Q reduction site. The γ-lactone head group is separated by a hydrophobic linker from the hydroxy-bis-tetrahydrofuran group, which is located approximately in the middle of the molecule. Although the lactone head group bears little resemblance to ubiquinone, it binds to the Q reduction site. Instead of a hydrogen bond, it forms a hydrophobic contact with the tyrosine residue ($\mathrm{Tyr}108_{\mathrm{Mm}}^{\mathrm{Fs}2}$). The β1 β2 loop histidine ($\mathrm{His}59_{\mathrm{Mm}}^{\mathrm{FS}2}$) is close to the ester bond. Further interactions of the head group involve $\mathrm{Met}152_{\mathrm{Mm}}^{\mathrm{FS}2}$ and $\mathrm{Met}70_{\mathrm{Mm}}^{\mathrm{FS}7}$. The distance of the lactone head group and the central hydroxy-bis-tetrahydrofuran group is such that the latter group can bind to the charged sector of the Q tunnel. The two hydroxyl groups in position 13 and 22 bind to glutamic acid residues ($\mathrm{Glu}24_{\mathrm{Mm}}^{\mathrm{ND}1}$ and $\mathrm{Glu}204_{\mathrm{Mm}}^{\mathrm{ND}1}$) of ND1.

## 7. Binding of a Synthetic Anti-Cancer Compound

A recent high-resolution structure revealed the binding mode of a synthetic anti-cancer agent to mammalian complex I. In contrast to the classical complex I inhibitors described above, this compound does not bind to the Q reduction site but rather blocks the entrance of the Q tunnel (Figure 3D). The compound IACS-2858 (Figure 1E) consists of a five-ring skeleton with four aromatic rings and one nonaromatic ring flanked on each end by a methylsulfonyl and a trifluoromethoxy group, respectively. The compound is inserted into the Q tunnel with the methylsulfonyl group in front. The terminal phenyl ring and the trifluoromethoxy group are outside of the tunnel. The methylsulfonyl group is bound in a network of polar and hydrogen-bonding interactions with $\mathrm{Arg}34_{\mathrm{Mm}}^{\mathrm{ND}1}$ and $\mathrm{Gln}32_{\mathrm{Mm}}^{\mathrm{ND}1}$. The 2-pyridone ring is bound by $\mathrm{Arg}87_{\mathrm{Mm}}^{\mathrm{FS}7}$ and part of a remarkable π-stacking arrangement with three aromatic residues ($\mathrm{Trp}56_{\mathrm{Mm}}^{\mathrm{FS}7}$, $\mathrm{Phe}224_{\mathrm{Mm}}^{\mathrm{ND}1}$, $\mathrm{Tyr}228_{\mathrm{Mm}}^{\mathrm{ND}1}$). This remarkable “cork in bottle” mechanism [45] is not only important for the development of pharmacological agents. Since the blockade of the tunnel entrance by the inhibitor leads to a complete loss of activity, a much-discussed alternative access route to the active site can also be excluded.

## 8. Conclusions

The binding of Q and inhibitors to respiratory complex I is now known in considerable detail. The three compounds from biological sources discussed here inhibit Q reduction by complex I because they occupy and block the Q-binding sites in the active site and in the access pathway, connecting it with the membrane (Movie S1). Binding of rotenone and piericidin in the Q reduction site close to the FeS cluster N2 involves the same residues that are also responsible for binding of the Q head group. On the other hand, the interaction of the lactone head group of acetogenin is less similar to that of ubiquinone. In exchange it has a pronounced interaction with charged residues in the central sector of the long Q access pathway. Remarkably, rotenone and piericidin can occupy additional binding sites in this segment of the Q tunnel. They thus reproduce an important property of Q which can also bind with its head group in this area. The inhibition mechanism of the synthetic anti-cancer agent IACS-2858 differs in that the tunnel entrance is blocked, but no binding occurs in the Q reduction site.

## 9. Materials and Methods

Structural data were displayed using either PyMOL 2.5.2 (Schrödinger, Inc., New York, NY, USA) [75] or UCSF ChimeraX 1.4 (Resource for Biocomputing, Visualization, and Informatics, San Francisco, CA, USA) [76]. The Q tunnel was calculated using the Caver 3.0 PyMol plugin (Human Computer Interaction Laboratory and Loschmidt Laboratories, Brno, Czech Republic) [77] for PyMOL (starting point conserved Tyr144 in *Y. lipolytica* NDUFS2, PDB ID 6RFR, probe radius 1.3 Å).

## Figures and Tables

**Table 1:** Details of structures and residues shown in Figure 2 and Figure 3.

Species		*M. musculus (Mm)*	*B. taurus (Bt)*	*O. aries (Oa)*	*Y.lipolytica (Yl)*	*T. thermophilus (Tt)*
PDB ID		6ZTQ,7PSA,7B93	7QSK	6ZKN	6RFR,7O6Y	6I0D
NDUFS2	His	His 59	His 59	His 59	His95	His38
	Tyr	Tyr108	Tyr108	Tyr108	Tyr144	Tyr87
	Met/Val	Met152	Met152	Met152	Met188	Val131
	Thr/Ser	Thr156	Thr156	Thr156	Ser193	Thr135
	Asp	Asp160	Asp160	Asp160	Asp196	Asp139
	Phe_1_	Phe167	Phe167	Phe167	Phe203	Phe146
	Phe_2_/Leu	Phe168	Phe168	Phe168	Leu204	Phe147
NDUFS7	Trp/Pro	Trp56	Trp46	Trp46	Trp77	Pro38
	Met_1_	Met69	Met59	Met59	Met90	Met50
	Met_2_	Met70	Met60	Met60	Met91	Met51
	Val/Ile	Val75	Val75	Val75	Ile106	Val67
	Phe	Phe76	Phe76	Phe76	Phe107	Phe68
	Arg	Arg78	Arg77	Arg77	Arg108	Arg77
ND1	Glu_1_	Glu24	Glu24	Glu24	Glu26	Glu35
	Gln	Gln32	Gln32	Gln32	Gln34	Gln43
	Arg_1_	Arg34	Arg34	Arg34	Arg36	Arg45
	Glu_2_	Glu204	Glu204	Glu202	Glu208	Glu225
	Phe/Gln	Phe224	Phe224	Phe224	Phe228	Gln245
	Tyr	Tyr228	Tyr228	Tyr228	Tyr232	Tyr249
	Arg_2_	Arg274	Arg274	Arg274	Arg297	Arg294
ND4	Arg	Arg142	Arg142	Arg142	Arg162	Arg143
	Lys	Lys206	Lys206	Lys206	Lys221	Lys204
	Trp	Trp215	Trp215	Trp215	Trp230	Trp213

## Data Availability

Data sharing not applicable.

## Supplementary Materials

The following supporting information can be downloaded at: https://www.mdpi.com/article/10.3390/ph15091088/s1, Figure S1: Rotenone bound to complex I; Movie S1: Molecules bound in the Q tunnel of complex I.
