# Supplementary material for: Binding of Natural Inhibitors to Respiratory Complex I
Source: Pharmaceuticals (Basel). 2022 Aug 31;15(9):1088. doi: 10.3390/ph15091088 (PMC9503403; doi:10.3390/ph15091088)
Supplement: Supplementary file 1 [file pharmaceuticals-15-01088-s001.zip › pharmaceuticals-1837598-Supplementary Materials_revised.pdf]

Supplementary Materials

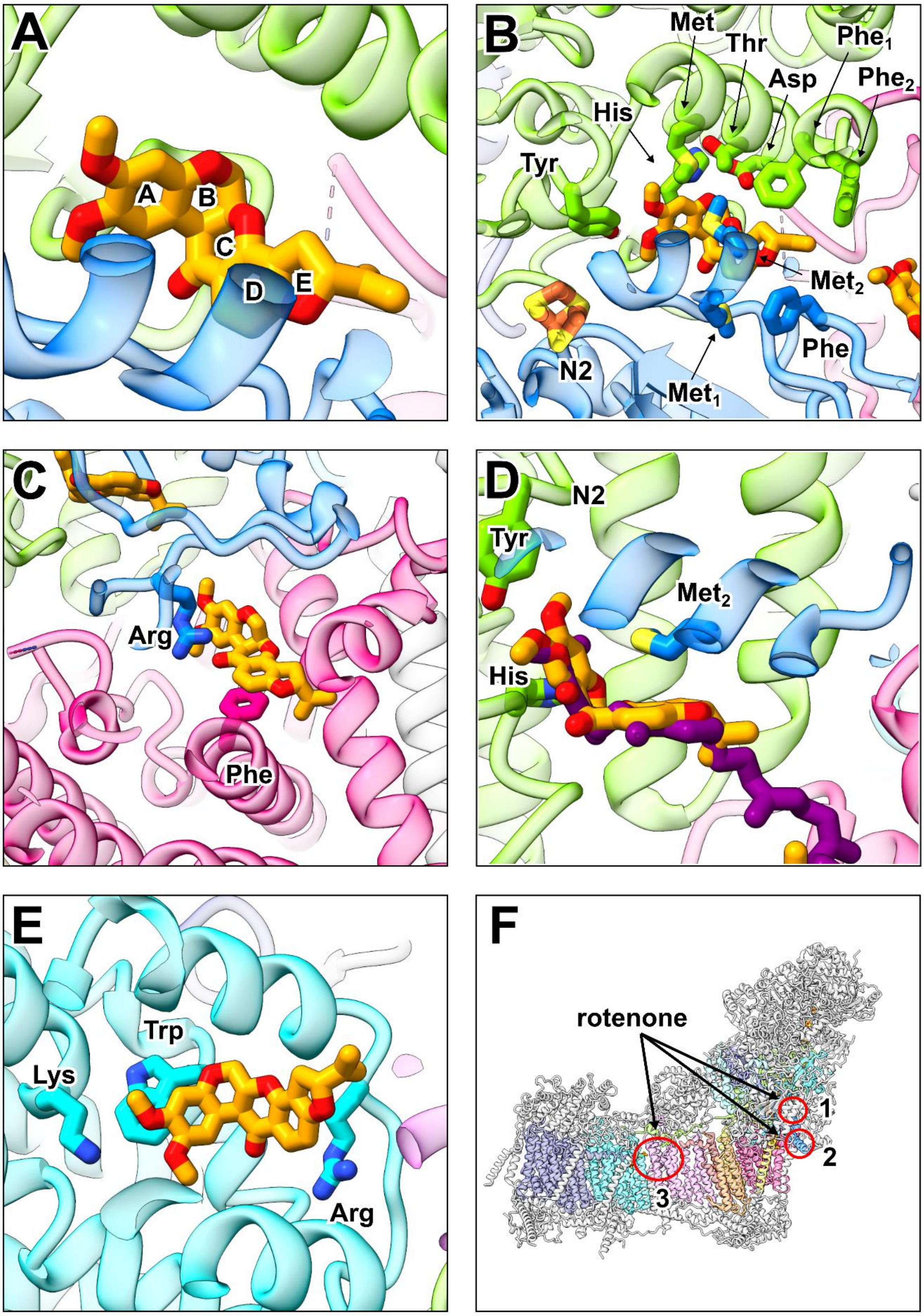

Supplementary Figure S1. Rotenone binding sites in complex I

Binding sites of rotenone in complex I (PDB ID 6ZKN); note that direction of view is changed as compared with Figs. 2, 3; for residue numbering see Table 1. (A, B) two detail views of rotenone in site 1 close to cluster N2, (C) detail view of rotenone bound to site 2 close to the exit of the Q tunnel, (D) overlay of rotenone in site 1 with Q10 (purple) (PDB ID 7QSK), (E) binding of rotenone at site 3 in the membrane arm, (F) the three rotenone binding sites in complex I (ND5 (dark blue), ND4 (cyan), ND2 (violet), ND4L (red), ND6 (orange), ND3 (yellow)) (ND1 (pink), NDUFS2 (green), NDUFS7 (blue)), shown in panels B (site 1), C (site 2), E (site 3).

Movie S1. Q and Inhibitors in the tunnel

Q molecules in the Q reduction site close to FeS cluster N2 (7QSK) and in the hydrophilic region (6RFR) of the Q-tunnel. In the following sequence, the positions of piericidin A (6ZTQ), rotenone (6ZKN), acetogeneine/compound 1 (7PSA) and the synthetic anti-cancer agent IACS-2858 (7B93) are shown. The residues depicted are the same as in Figures 2 and 3 for each structure respectively.
